# Denitrifier Community in the Oxygen Minimum Zone of a Subtropical Deep Reservoir

**DOI:** 10.1371/journal.pone.0092055

**Published:** 2014-03-24

**Authors:** Zheng Yu, Jun Yang, Lemian Liu

**Affiliations:** 1 Aquatic EcoHealth Group, Key Laboratory of Urban Environment and Health, Institute of Urban Environment, Chinese Academy of Sciences, Xiamen, P. R. China; 2 University of Chinese Academy of Sciences, Beijing, P. R. China; University of California, Merced, United States of America

## Abstract

Denitrification is an important pathway for nitrogen removal from aquatic systems and this could benefit water quality. However, little is known about the denitrifier community composition and key steps of denitrification in the freshwater environments, and whether different bacteria have a role in multiple processes of denitrification reduction. In this study, quantitative PCR, quantitative RT-PCR, clone library and 454 pyrosequencing were used together to investigate the bacterial and denitrifier community in a subtropical deep reservoir during the strongly stratified period. Our results indicated that the *narG* gene recorded the highest abundance among the denitrifying genes (2.76×10^9^ copies L^−1^ for DNA and 4.19×10^8^ copies L^−1^ for RNA), and the lowest value was *nosZ* gene (7.56×10^5^ copies L^−1^ for DNA and undetected for RNA). The RNA: DNA ratios indicated that *narG* gene was the most active denitrifying gene in the oxygen minimum zone of Dongzhen Reservoir. Further, α-, β- and γ- Proteobacteria were the overwhelmingly dominant classes of denitrifier communities. Each functional gene had its own dominant groups which were different at the genus level: the *narG* gene was dominated by *Albidiferax*, while *nirS* gene was dominated by *Dechloromonas*. The main OTU of *nirK* gene was *Rhodopseudomonas palustris*, but for *norB* and *nosZ* genes, they were *Bacillus* and *Bradyrhizobium*, respectively. These results contribute to the understanding of linkages between denitrifier community, function and how they work together to complete the denitrification process. Studies on denitrifier community and activity may be useful in managing stratified reservoirs for the ecosystem services and aiding in constructing nitrogen budgets.

## Introduction

Nitrogen (N), the fourth most abundant element (after oxygen, carbon and hydrogen) in microorganisms, is essential for the synthesis of nucleic acids and proteins [1]. It is commonly found as amine or amide groups in organic matter but is readily oxidized or reduced and thus, has an additional significance in aquatic systems as both an electron acceptor and a donor for energy metabolism [2]. All living organisms require a mass of nitrogen. Depending on the life form, nitrogen constitutes approximately 14% of the weight of a microbial cell [3]. Even though nitrogen is the overwhelmingly abundant element in the atmosphere, nitrogen gas (N_2_) is virtually inert. Most organisms cannot fix nitrogen but rather obtain their nitrogen directly as NH_4_^+^ (or organic nitrogen) from the environment or from the reduction of NO_3_^−^ to NH_4_^+^ through assimilatory nitrate reduction [4]. However, anthropogenic influences on the biogeochemistry of nitrogen for instance, combustion of fossil fuels, production of nitrogen fertilizers, cultivation of nitrogen-fixing legumes, and other actions have resulted in major changes in the earth's nitrogen cycle [5]. Whilst the production and industrial use of artificial nitrogen fertilizers worldwide has enabled humankind to greatly increase food production, consequently it has also led to a host of environmental problems, especially potential eutrophication of freshwater and coastal ecosystems [6].

Denitrification is a dissimilatory process in which oxidized nitrogen is used as an alternative electron acceptor for energy production when oxygen is limiting [7]. This process in the ocean consists of four reaction steps in which denitrifiers respire nitrate (NO_3_^−^) sequentially to nitrite (NO_2_^−^), nitric and nitrous oxides (NO, N_2_O) and finally to N_2_ [8]. It plays an important role in nitrogen transformation and removal and is responsible for the return of fixed nitrogen back to the atmosphere [9]. Alexander et al [10] pointed out that more than 75% of the anthropogenic nitrogen (N) entering watersheds were lost along landscape flow paths before reaching the oceans by heterotrophic denitrification. Thus, heterotrophic denitrification is an important microbial process in terms of water quality [11]. However, denitrification is also a large source of nitrous oxide (N_2_O) emissions in terrestrial and aquatic ecosystems, because the N_2_O is a potent greenhouse gas that contributes to climate change and stratospheric ozone destruction [12]. Thus only when the nitrate reduction process proceeds down to the production of N_2_ will it benefit the water-atmosphere systems.

Lakes and reservoirs are now being recognized as an important location for the removal of nitrogen from the lacustrine environment due to long water residence times and high biological activity in the oxygen minimum zone (OMZ) [13]. The oxygen concentrations in the OMZ are low enough to induce anaerobic metabolism and 20% to 40% of the total loss of nitrogen is estimated to occur in these zones [14]. In fact, denitrification is a facultative anaerobic microbial process [15]. The denitrifiers are known to belong to more than 50 genera of phylogenetic bacteria, including members of the Proteobacteria, Firmicutes, Actinobacteria, Bacteroides, and Planctomyces [16]. As many denitrifying organisms are unable to produce the entire suite of enzymes to complete the denitrification reduction processes, other organisms within the community are required to cooperate to complete the process [17]. Molecular techniques targeting functional genes that code for the enzymes in nitrate reduction processes have been established as molecular markers [18]. Over the past decades, *narG* (encoding the membrane-bound nitrate reductase), *nirS* (encoding the cytochrome cd1-containing nitrite reductase), *nirK* (encoding the Cu-containing nitrite reductase), *norB* (encoding nitric oxide reductase) and *nosZ* (encoding the nitrous oxide reductase) genes had been widely used to describe denitrifier communities [19]–[22].

In the present study, the distributions of denitrifier communities were investigated in a typical subtropical deep reservoir, where strong thermal stratification and a clear anaerobic layer marked the autumn season in the bottom water column. Clone library, quantitative PCR, quantitative RT-PCR and 454 pyrosequencing were used together to investigate the bacterial and denitrifier abundance, activity and composition in the freshwater OMZ. We aimed to explore the main steps of denitrification in a freshwater environment, and identify whether different bacteria have different roles in the denitrification reduction processes. This research aims to improve understanding of the linkages between denitrifier community structure, function and how they work together to complete the denitrification process.

## Materials and Methods

### Ethics statement

No specific permissions were required for these activities. Informed consent was obtained from all participants and this field study did not involve endangered or protected species.

### Study sites, sample collection and nucleic acid extraction

Dongzhen Reservoir (25°28′–25°30′N, 118°54′–118°59′E) is a seasonally stratified reservoir located in southeast China. Samples were collected within the main lacustrine zone close to the dam in October 2011 when water was fully stratified. We characterized the water temperature, dissolved oxygen (DO), chlorophyll a (Chl-a), pH, and electrical conductivity (EC) of the water column at 1-m intervals using a multi-parameter water quality analyzer (Hydrolab DS5, Hach Co., USA). The water oxygen minimum zone was identified in the benthic zone (24–36 m) where the oxygen concentration was as low as 0.2 mg/L. Total nitrogen (TN) was determined using a Shimadzu TOC-VCPH analyzer (Shimadzu, Japan). Total phosphorus (TP) was analyzed by spectrophotometry after digestion. Ammonium nitrogen (NH_4_-N), nitrite and nitrate nitrogen (NO_x_-N) and phosphate phosphorus (PO_4_-P) were measured with a Lachat QC8500 Flow Injection Analyzer (Lachat Instruments, USA). Three 1 litre replicate water samples were filtered immediately through a 0.22-μm polycarbonate membrane (47 mm diameter, Millipore, USA). The membranes were frozen at −80°C prior to further molecular analyses. All sampling and instrument casts were made from a station over the deepest area of the reservoir (36 m). Total DNA was extracted directly from the membranes using the FastDNA spin kit (Bio101, USA) according to the manufacturer's instructions. Purified DNA was dissolved in 50 μl ddH_2_O and stored at −20°C until use. Total RNA was extracted from the membranes using the E.Z.N.A. total RNA kit II (Omega Bio-Tek, USA) following the manufacturer's instructions. After the extraction procedure, RNA was transcribed into complementary DNA using the Takara OneStep RT-PCR kit Version 2.0 following the manufacturer's instructions. Reverse transcription was performed as a 15 min reaction at 37°C terminated by 5 sec incubation at 95°C.

### Clone library and sequence analysis

The 16 S rRNA gene and five denitrifying genes (*narG, nirS, nirK, norB* and *nosZ*) were amplified from extracted DNA using the primers following Table 1 [23]–[26]. We pooled the three replicates of PCR products from each of the denitrifying functional genes and 16 S rRNA gene for clone libraries analysis. Agarose gel electrophoresis of the 50 μl PCR products were performed prior to purification (QIAquick Gel Extraction Kit, Qiagen). Purified PCR products were ligated into the pMD18-vector (Takara, Japan) and transformed into *Escherichia coli* DH5α competent cells (Takara, Japan). Positive clones were grown in Luria-Bertan (LB) medium overnight at 37°C. Plasmids containing target gene fragments were identified by agarose gel electrophoresis and sequenced using an automatic capillary sequencer (ABI3730, USA).

**Table 1:** PCR Primers used in this study.

Target gene	Primer	Primer sequence	Annealing temperature (°C)	Amplicon size (bp)	Reference
Clone library					
16 S rRNA	341F	CCTACGGGNGGCWGCAG	53	465	[23]
	805R	GACTACHVGGGTATCTAATCC			
*NarG*	1960F	TA(CT)GT(GC)GGGCAGGA(AG)AAACTG	55	691	[24]
	2650R	TTYTCRTACCABGTBGC			
*NirS*	cd3aF	GT(C/G) AAC GT(C/G) AAG GA(A/G) AC(C/G) GG	60	426	[25]
	R3cd	GA(C/G) TTC GG(A/G) TG(C/G) GTC TTG A			
*NirK*	F1aCu	ATCATGGT(C\G)CTGCCGCG	53	473	[25]
	R3Cu	GCCTCGATCAG(A/G)TTGTGGTT			
*NorB*	2F	GACAARHWVTAYTGGTGGT	52	428	[26]
	7R	CCRTGGSTRWARWARTTSAC			
*NosZ*	F	CG(C/T)TGTTC(A/C)TCGACAGCCAG	58	454	[25]
	1622R	CG(G/C)ACCTT(G/C)TTGCC(C/G)T(T/C)GCG			
Quantitative PCR					
16S rRNA	341F	CCTACGGGNGGCWGCAG	60	175	[27]
	515R	ATTCCG CGG CTG GCA			
*NarG*	1960m2F	TA(CT)GT(GC)GGGCAGGA(AG)AAACTG	60	100	[27]
	2050m2R	CGTAGAAGAAGCTGGTGCTGTT			
*NirS*	2F	TACCACCC(C/G)GA(A/G)CCGCGCGT	60	165	[19]
	3R	GCCGCCGTC(A/G)TG(A/C/G)AGGAA			
*NirK*	876F	ATYGGCGGVAYGGCGA	60	165	[28]
	1040R	GCCTCGATCAGRTTRTGGTT			
*NorB*	2F	GACAARHWVTAYTGGTGGT	60	394	[26]
	6R	TGCAKSARRCCCCABACBCC			
*NosZ*	1F	WCSYTGTTCMTCGACAGCCAG	60	260	[29]
	1R	ATGTCGATCARCTGVKCRTTYTC			

### Quantitative PCR and RT-PCR

The successfully sequenced plasmids from clone libraries were extracted using the MiniPrep kit (Qiagen, Germany) and the plasmid concentrations were determined by spectrophotometry using a BioPhotometer (Eppendorf, Germany). Standard primer sets were prepared from linearized plasmid serial dilutions containing between 10^2^ and 10^10^ of 16 S rRNA gene and denitrifying gene copies calculated directly from the concentration of extracted plasmid. Standard curves were generated by plotting the threshold cycle values versus log10 of the gene copy numbers. The amplification efficiency (*E*) was estimated using the slope of the standard curve through the following formula: *E* = (10^−1/slope^)−1. The efficiency of the PCR was between 95% and 105% in this study. The relation efficiencies of the standard curve with r^2^ were ≥0.99. The quantitative PCR and quantitative RT-PCR assays were carried out in a volume of 20 μl including 10 μl SYBR Premix Ex Taq, 0.4 μl of ROX Reference Dye II, 0.5 μM of each primer [19], [26], [27], [28], [29], 2 μl of total DNA or cDNA, and 6.8 μl RNase-free water. The thermocycling steps of real-time PCR were run according to the manufacturer's instructions (Takara, Japan). All the measurements were performed and verified in triplicate. Real-time PCR with standard curves was used as the absolute quantification to calculate the concentrations of 16 S rRNA gene and denitrifying functional genes. After evaluation of the analysis parameters, the relation efficiencies for the standard curves were: r^2^ = 0.992 for 16 S rRNA, 0.990 for *narG*, 0.993 for *nirS*, 0.994 for *nirK*, and 0.992 for *nosZ*, respectively. The efficiency of the PCR amplification was 103.9% (16 S rRNA), 102.4% (*narG*), 102.0% (*nirS*), 101.8% (*nirK*) and 101.3% (*nosZ*), respectively. As a useful approach to estimate the gene activity of the bacterial and denitrifier community, we also compared the RNA to DNA ratios of 16 S rRNA and denitrifying genes.

### 454 pyrosequencing

In order to independently verify our clone library results, 454 pyrosequencing was used to facilitate an in-depth investigation of the bacterial community composition. PCR was performed using 454 sequencing adaptor-linked primers flanking the 16 S rRNA gene V3–V5 region [30]. The 16 S rRNA genes were PCR amplified using broad range bacterial primers. The primers: 357F (CCTACGGGAGGCAGCAG) and 926R (CCGTCAATTCMTTTRAGT) were complemented with 454 adapters and sample specific barcodes. The 50-μl PCR mixture contained 1 μl of the primer set (10 μm each), 0.125 μl (5 U/μl) of Ex Taq DNA polymerase (Takara Bio, Otsu, Japan), 2.5 μl of Ex Taq buffer (20 mM MgCl_2_), 2 μl of deoxyribonucleotide triphosphate mixture (2.5 mM each, Takara Bio, Otsu, Japan) and 50 ng of DNA template under the following running conditions: initial denaturation at 94°C for 4 min, 25 cycles of 30 s at 94°C, 45 s at 50°C, 1 min at 72°C, and a final elongation step for 8 min at 72°C. PCR products were confirmed using agarose gel electrophoresis and these were subsequently isolated from the gel and purified using a GeneJET gel extraction kit (Thermo Fisher Scientific). Sequencing reactions were performed by utilizing a Roche 454 FLX instrument (Roche, Indianapolis, IN) with Titanium reagents at Personal Biotechnology Company (Shanghai, China). Sequences were processed by using MOTHUR v.1.20.1 [31]. Briefly, any sequences with a length <200 or >1000, mean quality <25, ambiguous bases >1, homopolymer length >6, maximum primer mismatch >0 were removed from further analysis. Sequence reads were clustered to give similarity-based OTUs using a cluster database at high identity with tolerance (cd-hit) at minimum sequence identity set of 97% [32]. The main phylum level data of bacterial communities were used to compare with the clone library analysis results.

### Data analysis

For the clone library, the operational taxonomic unit (OTU) accumulation curve, abundance-based coverage estimator (ACE) and Chao1 estimator were calculated in MOTHUR v.1.20.1 [31]. The sequences with similarities greater than 97% were grouped in one OTU. Each sequence was compared with sequences available in GenBank databases using BLAST. Nucleotide Blast was used for the 16 S rRNA gene analysis and for denitrifying genes to search the translated nucleotide database using the translated denitrification nucleotide. The closest relatives were identified for phylogenetic analysis. The identities of our denitrifying fragments ranged from 76% to 99% (average identity of 88.7%) at the nucleotide level. Denitrifier abundances at both genus and phylum levels were measured with 85% and 65% sequence similarity cutoffs [33]. The sequences were realigned and manually edited with the ClustalX aligner and the phylogenetic analyses were performed with the MEGA5.0 software package using the neighbor-joining and maximum-likelihood method. The Jukes-Cantor model numbers at the branches show the bootstrap percentages (above 50% only) after 1000 replications of bootstrap sampling [34].

### Nucleotide sequence accession number

All sequence data from this study have been deposited in the public NCBI database (http://www.ncbi.nlm.nih.gov/) under the accession number KF978754-KF978818 and SRR1069588.

## Results

### Gene abundance, diversity and activity

**Figure 1:**
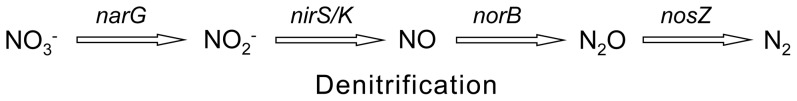
Scheme for nitrogen transformation from NO_3_^−^ to N_2_ by denitrification. Genes encoding enzymes that mediate the denitrification steps include those for nitrate reductase (*narG*), nitrite reductase (*nirS*/*nirK*), nitric oxide reductase (*norB*) and nitrous oxide reductase (*nosZ*).

**Figure 2:**
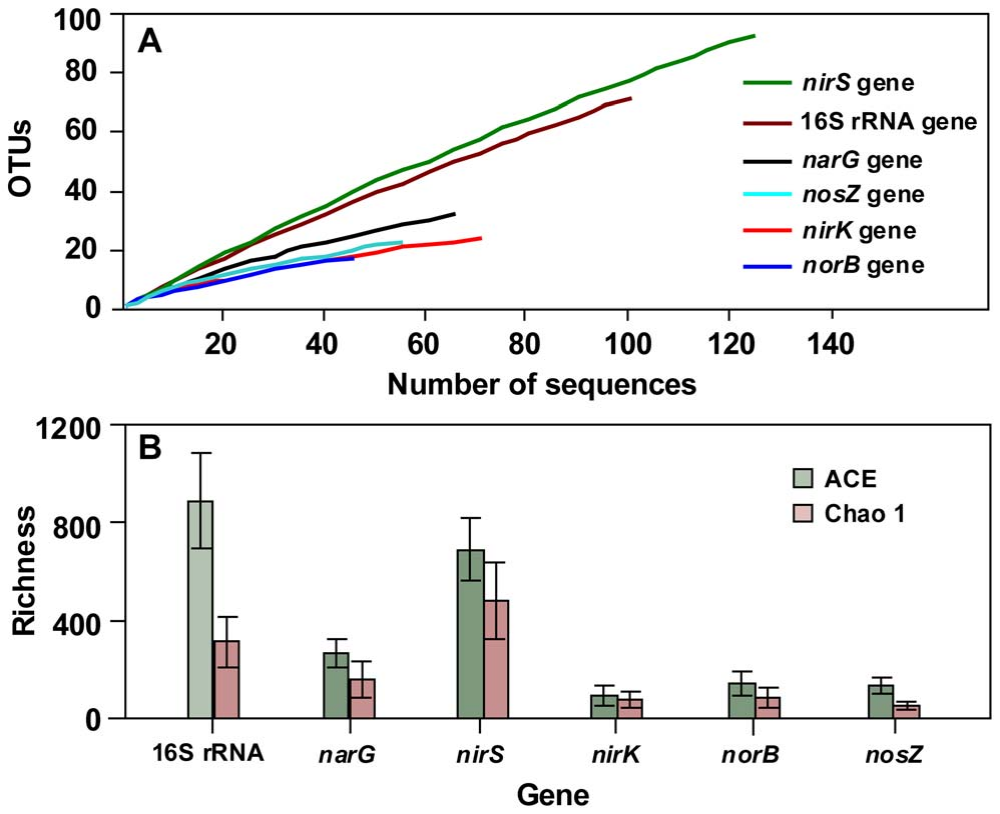
(A) Rarefaction curves of OTUs, which were defined at 97% sequence similarity for the 16 S rRNA gene and denitrifying gene sequences. (B) Richness estimates (ACE, Chao1) for six different clone libraries with MOTHUR at 97% similarity level.

**Figure 3:**
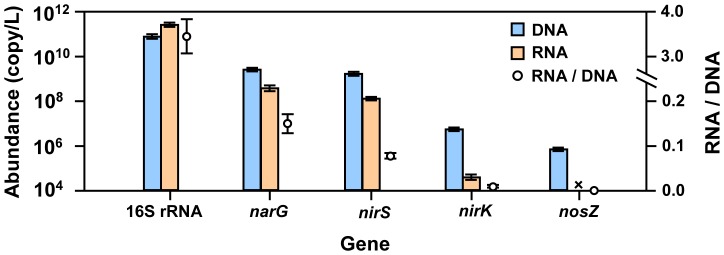
Number of 16S rRNA and denitrifying gene copies per litre water and RNA: DNA ratio of five genes in Dongzhen Reservoir. Error bars indicate standard errors of the three replicates samples with three triplicate qPCR reactions. “x” indicate undetected data from quantitative RT-PCR.

Six clone libraries including 16 S rRNA, *narG*, *nirS*, *nirK*, *norB* and *nosZ* genes were successfully constructed (Figure 1). Based on the 99 (16 S rRNA), 64 (*narG*), 124 (*nirS*), 69 (*nirK*), 43 (*norB*) and 53 (*nosZ*) sequenced clones, 71, 31, 93, 24, 17 and 22 OTUs were obtained for each gene at the 97% similarity level, respectively (Figure 2A). The order of the denitrifying genetic richness (number of estimated genotypes based on ACE and Chao1) was as follows: *nirS* > *narG* > *nosZ* > *norB* > *nirK* for ACE and *nirS* > *narG* > *nirK* > *norB* > *nosZ* for Chao1 (Figure 2B). A standard curve was used as a reference to calculate the concentrations of environmental DNA/RNA samples. The *narG*, *nirS*, *nirK*, and *nosZ* gene copy numbers were lower than the 16 S rRNA gene at both DNA and RNA levels (Figure 3). The *norB* gene was not detected in our samples. The *narG* gene recorded the highest abundance among the denitrifying genes in OMZ, and its mean value was 2.76 × 10^9^ copies L^−1^ for DNA and 4.19 × 10^8^ copies L^−1^ for RNA, respectively. The lowest value of denitrifying gene was *nosZ*, its mean value was 7.56× 10^5^ copies L^−1^ for DNA and undetected for RNA. The order of the denitrifying gene abundance and activity was as follows: *narG* > *nirS* > *nirK* > *nosZ*. The ratio of ribosomal RNA to DNA can be a good indicator of cellular activity, RNA to DNA ratios (RNA: DNA) were calculated for each gene. The highest RNA: DNA ratios were in the 16 S rRNA (3.452±0.381), followed by decreasing activity in the *narG* (0.150±0.021), *nirS* (0.078±0.006), and *nirK* (0.007±0.001). This indicated that *narG* gene was the most active denitrifying gene in the oxygen minimum zone of Dongzhen Reservoir.

### Denitrifying community

**Figure 4:**
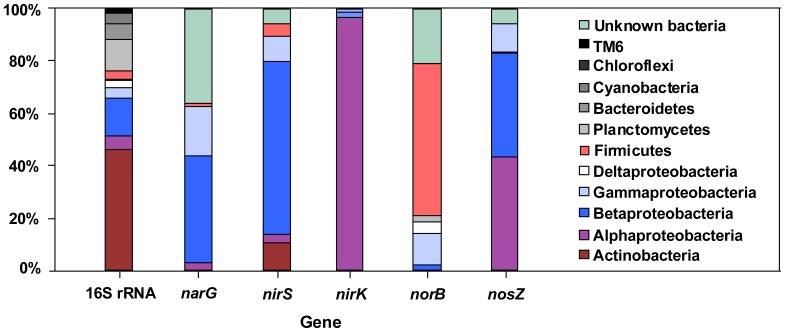
Relative abundance of different bacterial groups from 16 S rRNA gene and five denitrifying genes based on the clone library analysis.

**Figure 5:**
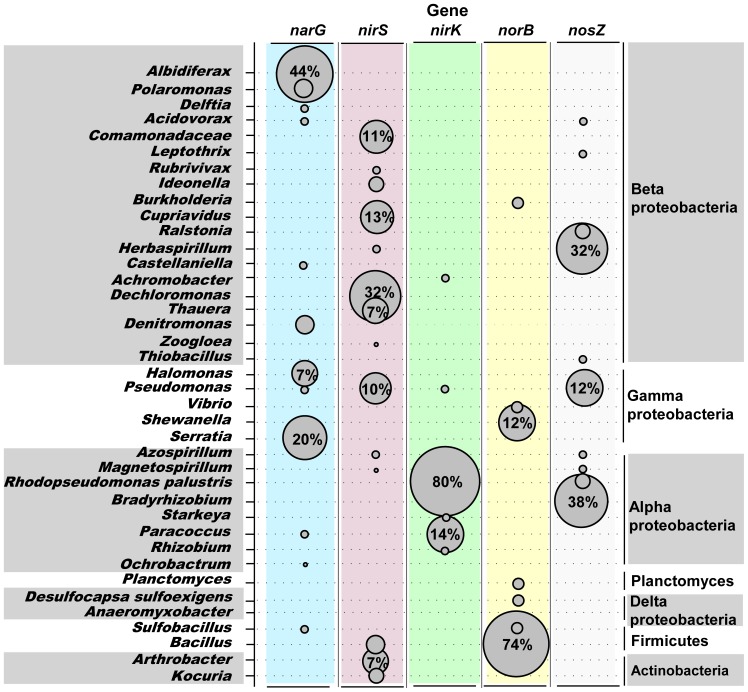
Relative abundance of different denitrifying genes at the genus (or species) level in Dongzhen Reservoir. Shown are OTUs assigned to the highest taxonomic level possible using a BLASTN in GenBank database. The circle size corresponds to the relative average abundance OTUs for each denitrifying gene.

Gene encoding for the membrane-bound nitrate reductase (*narG*) was distributed among taxonomically diverse bacteria, from α-, β-, γ- Proteobacteria and Firmicutes, and the most represented *narG* gene group was the β- Proteobacteria (40.6%). The amplified *nirS* fragments were distributed from α-, β-, γ- Proteobacteria, Actinobacteria and Firmicutes. About 66.1% of the clone library sequences were from the β- Proteobacteria. In contrast to the high richness of *nirS*, the Cu-containing enzyme (*nirK*) had 24 OTUs and α- Proteobacteria was the overwhelmingly dominant group which occupied 96.9% of the total sequences. The *norB* gene was found to be present in the bacteria of β-, γ-, δ- Proteobacteria, Planctomycetes and Firmicutes, and most likely due to the bacterial group of Firmicutes (occupied 58.1% of the total *norB* gene sequences). For the *nosZ* gene most bacterial taxa were distributed from α-, β-, γ- Proteobacteria. Strong support groups were observed for α- and β- Proteobacteria (43.2% and 39.6% respectively) (Figure 4). In order to conduct more detailed analysis in the denitrifying community composition, we organized all of the sequencing reads and assigned to the genus and species level. The denitrifier communities were found to be different at the genus/species level. For example, the *narG* gene was dominated by *Albidiferax* but the *nirS* gene was dominated by *Dechloromonas*. The main OTU of *nirK* gene was *Rhodopseudomonas palustris*, but for *norB* and *nosZ* genes, they were *Bacillus* and *Bradyrhizobium*, respectively. In total, 452 sequences were compared with the GenBank databases one by one, and there were very little two enzymes have been found to coexist in the same denitrifying organism. The same bacterial genus seldom had multi denitrifying genes except only a few genera such as *Pseudomonas*, *Herbaspirillum*, *Paracoccus* (Figure 5).

### Comparison of the microbial community composition obtained using pyrosequencing and clone library

**Figure 6:**
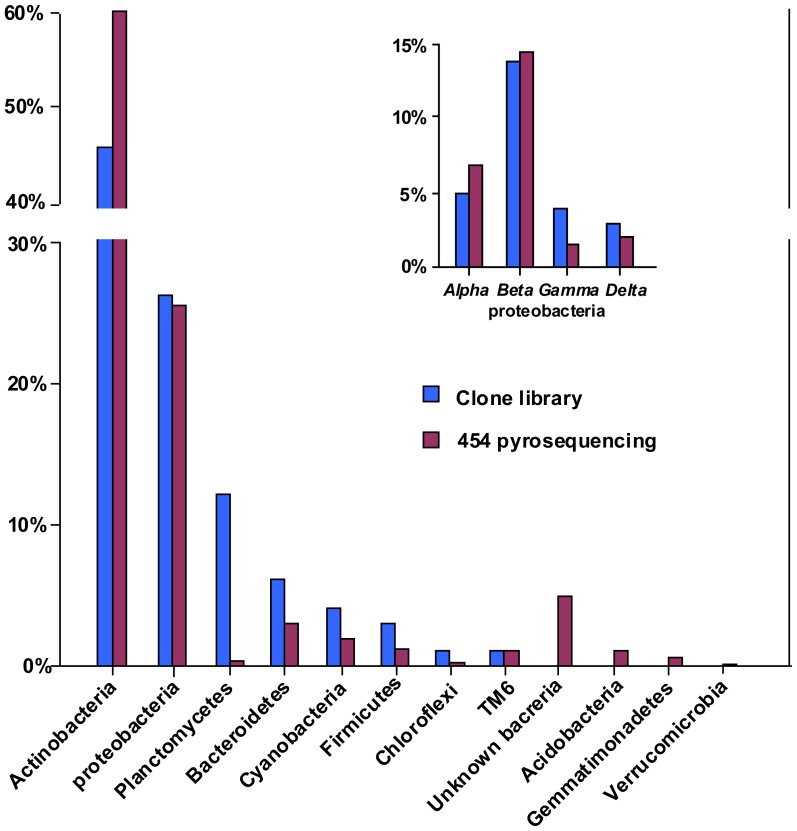
Bacterial community compositions at phylum level and Proteobacteria at class level revealed by clone library and 454 pyrosequencing.

At the phylogenetic phylum level, the predominant bacteria indicated by 454 pyrosequencing and clone library methods were roughly the same proportion (Figure 6). The two methods were both indicated that Actinobacteria was the overwhelmingly dominant bacterial phylum in Dongzhen Reservoir, and α-, β-, δ- and γ- Proteobacteria, Bacteroidetes, Cyanobacteria, Firmicutes and Chloroflexi were found with similar patterns. However, the pyrosequencing detected fewer sequences from the phylum Planctomycetes but more sequences from a broader range for the low abundance bacterial groups (for example, Acidobacteria, Gemmatimonadetes and Verrucomicrobia) compared with the clone library approach (Figure S1 and Figure S2).

## Discussion

### Nitrate and nitrite reduction as the dominant denitrification process

Denitrification often occurs in specific habitats where oxygen is limited, such as the oxic-anoxic interface of benthic sediments, or in the water column at the edge of suboxic or anoxic water masses in OMZs [35]. Denitrification has been proved to be an important pathway to reduce nitrate loads in aquatic ecosystems [1], [36]. Previous research has shown that different environmental conditions may favor organisms with different key denitrifying genes [2]. For instance, in estuarine wetland sites, the nitrate (*narG*) and nitrite (*nirS*) functional genes were relatively dominant during cold and warm seasons, respectively [37]. Further, in rice paddy field soil, the community structures based on *nirS* and *nirK* sequences were different among the sampling times and *nirK* was more abundant than *nirS* [38]. In the marine ecosystem, N is often a limiting resource and the relative importance of denitrifier communities differs along the environmental N gradient [15]. Our results indicated that in the freshwater OMZ, all five denitrifying genes were present and the *narG* and *nirS* genes were identified to be the dominant and diverse denitrification reduction genes. The RNA: DNA ratio of the *narG* gene was higher than those of three other denitrifying genes, suggesting that *narG* gene was the most active denitrifying gene in terms of nitrogen loss in the reservoir's OMZ. Further research is warranted to establish whether this is a universal phenomenon in other freshwater ecosystems. Normally, denitrification occurs when three conditions are satisfied: i) N sources for denitrification are available; ii) oxygen concentrations are reduced; iii) electron donors are available [39]. In the present study, the reservoir's OMZ met these conditions. The concentrations of nitrate (NO_3_^−^) and nitrite (NO_2_^−^) (Table 2) in our sampling sites were suitable for encoding microbial *narG* and *nirS* genes. Abundance of organic substances that can be used as electron donors for the heterotrophic denitrifiers exists in the reservoir's OMZ. Interestingly, the *nosZ* gene was detected from extracted DNA with an average copy number of 7.56 × 10^5^ L^−1^, but it was not detected at the RNA level. A possible explanation could be that the gaseous products of denitrification are the major biological pathway for N loss, then the gaseous intermediates (NO and N_2_O) would escape from the water to the atmosphere. Finally the nitrous oxide reduction process lacks N_2_O input as the electron acceptors in the OMZs [2]. The relative activity of the enzymes involved in denitrification may sometimes be affected by denitrifier composition, but in other cases nutrient limitation and environmental factors may be the dominant determinants of activity [15].

**Table 2:** Environmental variables in the oxygen minimum zone of Dongzhen Reservoir in October 2011.

Environmental variables	
Temperature (°C)	15.07±0.01
DO (mg L^−1^)	0.20±0.00
Chlorophyll a (μg L^−1^)	0.53±0.02
EC (mS m^−1^)	63.95±4.65
pH	6.765±0.045
TN (mg L^−1^)	8.641±0.197
NH_4_-N (mg L^−1^)	0.410±0.106
NO_x_-N (mg L^−1^)	0.883±0.027
TP (mg L^−1^)	0.037±0.002
PO_4_-P (mg L^−1^)	0.026±0.001

Values are mean ± SE (n = 3).

### Taxonomic diversity of denitrifiers

Our results clearly indicated that each denitrifying gene had its own dominant taxonomic group. The increasing numbers of sequenced bacterial genomes allow a comparison of the composition of denitrifying genes between different taxonomical groups [40]. For the *narG* gene, the dominant group showed highest similarity to the *Albidiferax* of β- Proteobacterium, which was originally isolated from sediment and is capable of reducing nitrate to nitrite [41]. Earlier studies of *narG* diversity in soil environments had previously identified sequences related to those from the Actinobacteria or Proteobacterial classes (α-, β- or γ- Proteobacteria) [42], which were consistent with the results of this study. Both *nirS* and *nirK* genes are two functionally-equivalent nitrite reductase enzymes which mediate reduction of nitrite to nitric oxide [2], [43]. However, previous research has shown that denitrifiers possess either *nirS* or *nirK*, and no strain is known to harbor both genes and enzymes so far [44], [45]. Our results revealed that the *nirS* and the *nirK* genes were different at the class level, *nirS* was dominated by β- Proteobacteria and *nirK* was overwhelmingly dominated by α- Proteobacteria (Figure 4 and Figure 5). The *norB* gene which encodes nitric oxide reductase catalyzes the reduction of NO to N_2_O, which represents an unusual reaction in biology, the formation of an N-N bond [20]. Our primer sets developed to detect the classes of *norB* genes and phylogenetic analysis identified nitrifier *norB* homologues as distinct from other denitrifier sequences. The majority of known denitrifiers which harbor *norB* genes are α- and β- subdivisions of the Proteobacteria [26], but the dominant group in the present study was Firmicutes. This phenomenon may be linked with environmental specificity [40]. The *nosZ* cluster has only been identified in the α-, β- and γ- Proteobacteria and no putative nitrous oxide gene cluster has been identified in Gram-positive or Archaebacteria [40]. In summary, our results indicated that the Proteobacteria were identified as the predominant denitrifier in the reservoir OMZ. These denitrifying bacteria were connected by their ability to grow chemolithotrophically at the expense of reduced nitrogen compounds. The distribution of abundant Proteobacterial denitrifiers in the water column may improve the self-purification ability of eutrophic reservoirs.

### Conclusions and implications for reservoir management

Denitrification is a stepwise reduction process involving four reaction steps: nitrate reduction, nitrite reduction, nitric oxide reduction, and nitrous oxide reduction [4], [24]. These key steps of the denitrification pathway are catalyzed by nitrite and nitrous oxide reductase [46]. In this study, we have shown a comprehensive view of the denitrifier abundance and composition in an oxygen minimum zone (OMZ) of a subtropical typical stratified reservoir. Proteobacteria were the overwhelmingly dominant phylum in the denitrifier community. The expression of five denitrifying functional genes had nine orders of magnitude of difference. Both *narG* and *nirS* gene groups were the dominant and active components of the OMZ denitrifier community with potential direct ties to the denitrification pathway. Previous researches have focused on denitrifying functional genes in marine and soil ecosystems [36]. Our research will enhance the understanding of the denitrification processes in freshwater ecosystems, in particular subtropical deep reservoirs. Normally, phosphorus is the most limiting nutrient in freshwater reservoirs [47], while nitrogen limitation is common in estuaries and oceans [15]. Denitrification is an immensely important process in removing nitrates and nitrites from inland waters before entering the coastal environments and driving eutrophication in those ecosystems. Thus understanding the denitrification in reservoirs along with pathways for nitrogen removal may benefit for water quality. Further research into denitrifier abundance, activity and community structure in freshwater OMZs is needed in order to formulate effective strategies for the water quality protections and improvements of drinking water sources.

## Supporting Information

Figure S1 **Neighbor-joining phylogenetic tree of 16 S rRNA gene sequences showing the positions of OTUs and taxonomic distribution.**
(TIF)

Figure S2 **Maximum-likelihood phylogenetic tree of 16 S rRNA gene sequences showing the positions of OTUs and taxonomic distribution.**
(TIF)
